# Identifying Brain Network Structure for an fMRI Effective Connectivity Study Using the Least Absolute Shrinkage and Selection Operator (LASSO) Method

**DOI:** 10.3390/tomography10100115

**Published:** 2024-09-30

**Authors:** Xingfeng Li, Yuan Zhang

**Affiliations:** 1 Department of Surgery & Cancer, Hammersmith Campus, Imperial College London, Du Cane Road, London W12 0HS, UK; 2 Key Laboratory of Language, Cognition and Computation of Ministry of Industry and Information Technology, School of Foreign Languages, Beijing Institute of Technology, 5 Zhongguancun South Street, Beijing 100081, China; yz@bit.edu.cn

**Keywords:** fMRI, model selection, system identification, brain imaging, visual cortex, effective connectivity, least absolute shrinkage and selection operator (LASSO)

## Abstract

**Background:** Studying causality relationships between different brain regions using the fMRI method has attracted great attention. To investigate causality relationships between different brain regions, we need to identify both the brain network structure and the influence magnitude. Most current methods concentrate on magnitude estimation, but not on identifying the connection or structure of the network. To address this problem, we proposed a nonlinear system identification method, in which a polynomial kernel was adopted to approximate the relation between the system inputs and outputs. However, this method has an overfitting problem for modelling the input–output relation if we apply the method to model the brain network directly. **Methods:** To overcome this limitation, this study applied the least absolute shrinkage and selection operator (LASSO) model selection method to identify both brain region networks and the connection strength (system coefficients). From these coefficients, the causality influence is derived from the identified structure. The method was verified based on the human visual cortex with phase-encoded designs. The functional data were pre-processed with motion correction. The visual cortex brain regions were defined based on a retinotopic mapping method. An eight-connection visual system network was adopted to validate the method. The proposed method was able to identify both the connected visual networks and associated coefficients from the LASSO model selection. **Results:** The result showed that this method can be applied to identify both network structures and associated causalities between different brain regions. **Conclusions:** System identification with LASSO model selection algorithm is a powerful approach for fMRI effective connectivity study.

## 1. Introduction

Since the discovery of blood oxygenation level-dependent (BOLD) functional MRI (fMRI) in 1990 [1], it has been extensively applied for studying brain functions. Both linear and nonlinear dynamic system modelling methods have been implemented for fMRI data analysis [2,3]. Dealing with the nonlinearity of the response of fMRI using dynamic system theory is a challenge due to the current limited availability of mathematical tools. In addition, because many biological systems and systems involving interactions between brain regions are available only as a black box, the relation between the system input (e.g., stimuli) and output (e.g., fMRI response) is not so derivable [4]. Wiener’s theory of nonlinear systems has been developed to address the brain system with fMRI methods [5]. Other methods such as the Granger causality (GC) [6] and the multivariate vector auto-regression (MVAR) model [7] have also been designed to study the causal relation between different brain regions from neuroimage, and there is increasing interest in applying MVAR for fMRI effective connectivity studies [8,9,10]. MVAR can be regarded as a special case of a nonlinear system identification method as the basis of a function using polynomials to model the system inputs and outputs. But this method does not consider the nonlinear dynamics in the fMRI response.

The brain neural population response is nonlinear; therefore, nonlinear modelling methods for modelling the response are better as it is close to reality. Different methods and models have been proposed to address this problem. For example, a phase portrait and fuzzy recurrence plot method [11] has been explored to model the fMRI response. Additionally, the Hopf model has been invented to study the link between the brain structure and dynamics in the whole brain [12]. A dynamical flexible inference for nonlinear embeddings has been created to model nonlinear brain dynamics [13]. However, it is difficult to infer the causality relationship between different brain regions using these methods.

System identification is one of the methods used to build mathematical models of dynamic systems using measurements of the input and output signals of the system [14]. This method includes finding the structure of the network and the associated parameters, although most current methods concentrate on identifying system parameters only, not on the structure of the network for fMRI effective connectivity studies. The main purpose of this paper was to improve the system identification method by using a statistical model selection algorithm to determine the structure of the network for brain region causality studies. Particularly, the least absolute shrinkage and selection operator (LASSO) model selection method [15,16] was applied to decide the effective connectivity for fMRI studies. Using a nonlinear system identification method with non-linear basis functions, such as polynomial or radial basis functions, a regularization step is required to reduce the number of predictors and smooth the estimation results. For fMRI studies, the current model selection is based on either L1 (sparsity) [17,18,19,20] or L2 norm (ridge regression) [21,22] and these methods have been mainly applied to functional connectivity studies [23,24,25]. Although the LASSO method has been adopted for causality studies using the electroencephalogram (EEG) method [26], the applicability of this method for nonlinear fMRI responses has not been studied.

Moreover, for fMRI effective connectivity studies with the MVAR method [27], this method does not include nonlinear terms and/or system inputs [28]. To overcome this limitation, the purpose of this study was to suggest a LASSO model selection algorithm [15,16] for effective connectivity based on the nonlinear system identification method. Specifically, a nonlinear system identification method with a model selection algorithm was applied to investigate the cause–effect relationship from different brain regions.

## 2. Materials and Methods

### 2.1. Subjects, Experimental Design, MRI Collection, and fMRI Image Pre-Processing

This is a retrospective study of a previous study [29], in which brain responses to external stimuli between healthy controls and amblyopic patients were compared. Subjects, experimental design, data collection, and fMRI image pre-processing can be found in the previous study [29]. Briefly, visual eccentricity and polar angle stimuli were adopted for the experimental design, and subjects viewed the stimuli monocularly. A 1.5-T (Magnetom; Siemens, Madison, WI, USA) scanner was adopted to collect structural and functional image data. The repetition time (TR) was 3 s for the fMRI data collection. The fMRI image resolution was 4 mm × 4 mm × 4 mm, and the data were obtained from the visual cortex. The fMRI was pre-processed with motion correction and the visual cortex boundaries were defined based on the responses. A Z-score method was employed to normalise the time series temporally. For each visual cortex region, a single voxel response was selected randomly to represent the fMRI response from the region. There were 128 image frames for the fMRI time series. The first 8 images were excluded from the analysis due to the instability of the magnetic fields at the beginning of the fMRI scan; therefore, only 120 fMRI time series were used. fMRI scans from one subject viewing the eccentricity and polar angle stimuli with the right eye were included, and only first-level (within each run) fMRI analysis was involved in this study.

### 2.2. Theory

Briefly, the discrete nonlinear dynamic brain can be described using a differential equation as follows [30]:

(1) $$\left\{\begin{array}{c} x(t+1)=f(x(t),u(t),\theta )\\ y(t)=g(x(t),\theta ) \end{array}\right.$$

where x(t) is the state variable, θ is the parameter, y(t) is the system output (fMRI response in this study), f and g are nonlinear functions, and u(t) is the system input (stimuli for task fMRI). For resting-state fMRI, u(t)=0. For the effective connectivity study, we need to identify the system connection structure and parameters in Equation (1).

**Figure 1:**
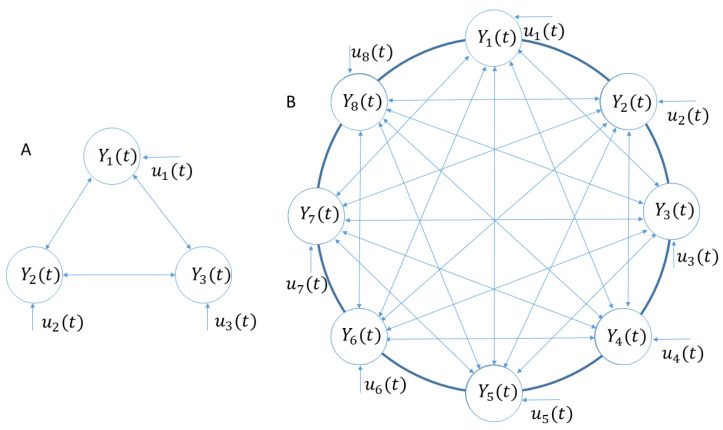
(**A**) An example of a three-connection network structure. (**B**) An example of an eight-connection network structure. Line arrow indicates the direction of information flow (causality).

Taking a 3-connection network (Figure 1A) as an example, it has a 3-connection network structure with responses from 3 brain regions. In the network, each region is connected to other regions mutually with an additional input. The inputs are used to represent the fMRI response due to the stimuli from the experimental design. Figure 1B shows another example with a larger network, which has an eight-connection network. For this network, $Y_{1}(t)$, $Y_{2}(t)$, $Y_{3}(t)$, $Y_{4}(t)$, $Y_{5}(t)$, $Y_{6}(t)$, $Y_{7}(t)$, and $Y_{8}(t)$ denote the fMRI time series from visual cortex regions of V1, V2, V3, Vp, V3a, V4v, V3b, and V5, respectively. For illustration purposes, we will only use these two network structures as shown in Figure 1, but this method can be applied to other network structures in the same way for brain effective connectivity studies.

To model the input–output relation of this nonlinear brain system as shown in Figure 1A, a polynomial kernel is adopted to approximate the nonlinear brain system. Using the first order auto-regression (AR) model, without taking into account nonlinear terms and neglecting the system inputs, for this 3-connection network (Figure 1A), we can write the first level fMRI causality analysis model as:

(2) $$Y_{1}(t)=a_{1}Y_{1}(t-1)+a_{2}Y_{2}(t-1)+a_{3}Y_{3}(t-1)+\varepsilon_{1}(t)$$

(3) $$Y_{2}(t)=b_{1}Y_{1}(t-1)+b_{2}Y_{2}(t-1)+b_{3}Y_{3}(t-1)+\varepsilon_{2}(t)$$

(4) $$Y_{3}(t)=c_{1}Y_{1}(t-1)+c_{2}Y_{2}(t-1)+c_{3}Y_{3}(t-1)+\varepsilon_{3}(t)$$

In Equations (2)–(4), $Y_{1}(t)$, $Y_{2}(t)$, and $Y_{3}(t)$ are the fMRI response from the brain regions at time point t. a, b, and c are the model coefficients, and ε is the model residual. For instance, from Equation (2), we can test the coefficient $a_{2}$ to study the influence from $Y_{2}(t-1)$ on $Y_{1}(t)$. This can be achieved by the F/T test. We adopted the F test to test whether $a_{2}$ is significantly larger than 0 or not. To achieve this, from Equation (2), we have a null hypothesis:

(5) $$H_{0}:a_{2}=0$$

The reduced model of Equation (2) can be written as:

(6) $$Y_{1}(t)=a_{1}Y_{1}(t-1)+a_{3}Y_{3}(t-1)+\varepsilon_{4}(t)$$

The Granger causality from $Y_{2}(t)$ to $Y_{1}(t)$ is quantified by the F test as:

(7) $$F_{1-\alpha }\left(q,N-k\right)=\frac{(RRSS-URSS)/q}{\left(URSS)/(N-k\right)}$$

where the RRSS denotes the Restricted Residual Sum of Squares (RRSS) as the residual sum of squares obtained from estimating the restricted model (i.e., from Equation (6), $RRSS=\varepsilon_{4}(t)⸱{\varepsilon_{4}(t)}^{T}$). URSS represents the unrestricted residual sum of squares (URSS) as the residual sum of squares obtained from estimating the unrestricted model (i.e., from Equation (2), $RRSS=\varepsilon_{1}(t)⸱{\varepsilon_{1}(t)}^{T}$). α is the level of significance. q is the degrees of freedom of the numerator, as we only test one parameter, i.e., q = 1 in this example. N−k is the denominator degree of freedom. In our case, the T test square is the F test, and the corresponding T test is:

(8) $$T_{N-k}^{2}=F(q,N-k)$$

with the denominator degree of freedom N−k. N is the sample size (length of fMRI time series), and k is the number of predictors in the reduced model, i.e., k=2 in this example.

### 2.3. Visual System

In this study, an eight-connection human visual system was used, and the connection network is displayed in Figure 1B. In the system, we adopted the polynomial basis function to approximate the nonlinear dynamic system. If we neglect these nonlinear interacting terms and high-order autoregression terms (using a 3-order autoregression term or below), the system output $Y_{1}(t)$ (fMRI response from V1 region) can be modelled as:

(9) $$\begin{array}{ll} Y_{1}\left(t\right)=a_{0}+a_{1} & Y_{1}\left(t-1\right)+a_{2}Y_{2}\left(t-1\right)+\cdots +a_{8}Y_{8}\left(t-1\right)+b_{1}Y_{1}\left(t-2\right)\\ & +b_{2}Y_{2}\left(t-2\right)+\cdots +b_{8}Y_{8}\left(t-2\right)+c_{1}Y_{1}\left(t-3\right)+c_{2}Y_{2}\left(t-3\right)\\ & +\cdots +c_{8}Y_{8}\left(t-3\right)+d_{1}u_{1}\left(t-1\right)+d_{2}u_{2}\left(t-1\right)+\cdots \\ & +d_{8}u_{8}\left(t-1\right)+e_{1}u_{1}\left(t-2\right)+e_{2}u_{2}\left(t-2\right)+\cdots +e_{8}u_{8}\left(t-2\right)\\ & +f_{1}u_{1}\left(t-3\right)+f_{2}u_{2}\left(t-3\right)+\cdots +f_{8}u_{8}\left(t-3\right)+g_{1}Y_{1}^{2}\left(t-1\right)\\ & +g_{2}Y_{2}^{2}\left(t-1\right)+\cdots +g_{8}Y_{8}^{2}\left(t-1\right)+h_{1}Y_{1}^{2}\left(t-2\right)\\ & +h_{2}Y_{2}^{2}\left(t-2\right)+\cdots +h_{8}Y_{8}^{2}\left(t-2\right)+i_{1}Y_{1}^{2}\left(t-3\right)+i_{2}Y_{2}^{2}\left(t-3\right)\\ & +\cdots +i_{8}Y_{8}^{2}\left(t-3\right)+j_{1}u_{1}^{2}\left(t-1\right)+j_{2}u_{2}^{2}\left(t-1\right)+\cdots \\ & +j_{8}u_{8}^{2}\left(t-1\right)+k_{1}u_{1}^{2}\left(t-2\right)+k_{2}u_{2}^{2}\left(t-2\right)+\cdots \\ & +k_{8}u_{8}^{2}\left(t-2\right)+l_{1}u_{1}^{2}\left(t-3\right)+l_{2}u_{2}^{2}\left(t-3\right)+\cdots +l_{8}u_{8}^{2}\left(t-3\right)\\ & +\varepsilon \left(t\right) \end{array}$$

There are 97 parameters that need to be estimated in Equation (9), i.e., 24 items from the AR of the fMRI response, 24 items from the system inputs, 24 nonlinear (square) responses, 24 nonlinear system input items, and $a_{0}$ is the intercept. The reason for excluding nonlinear interaction terms such as $Y_{2}(t-1)⸱Y_{3}(t-2)$ is because the effect is the combination of two different regions, which is not easy to interpret from the brain science viewpoint. Equation (9) can be written in matrix form as:

(10) $$Y=\beta_{0}+x_{i}^{T}\beta +\varepsilon$$

where Y is the $\left(n-AR\right)\times 1$ vector to represent the fMRI response $Y_{1}\left(t\right)$, n is the number of fMRI time frames, and AR is the maximum order of AR. $x_{i}$ is the (n−AR)×1 vector value at observation i, i.e., the data vector. $x_{1}=Y_{1}\left(t-1\right),x_{2}=Y_{2}\left(t-1\right),\cdots ,x_{96}=u_{8}^{2}\left(t-3\right)$. β is the coefficient vector of each term in Equation (9), $\beta_{0}=a_{0}$ is the intercept, and ε is the residuals.

For the causality within the seed region $Y_{1}$ (for the other regions, the same method can be applied), the least square solution of the equation is to minimise the mean square error objective function:

(11) $$\underset{\beta_{0},\beta }{\min}\left(\frac{1}{2N}\sum_{i=1}^{N}{\left(y_{i}-\beta_{0}-x_{i}^{T}\beta \right)}^{2}+\lambda \sum_{j=1}^{p}\left|\beta_{j}\right|\right)$$

where N=n−AR and p is the number of coefficients, i.e., p=97 in this study (including the intercept). λ is a positive regularisation parameter corresponding to one value of Lambda.

MATLAB (version 2022b) was employed for modelling the visual system using Equation (10) while minimising Equation (11). Function lassoglm.m from MATLAB was adopted to implement the LASSO model selection.

## 3. Results

**Figure 2:**
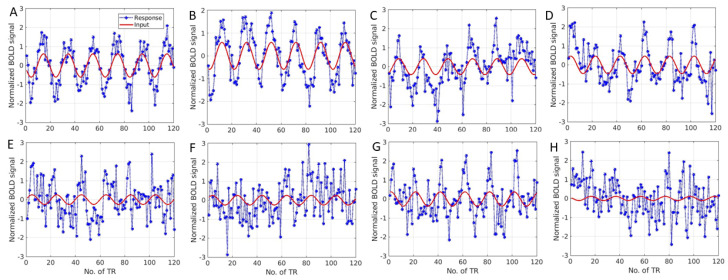
The fMRI time series from the eight-connection visual system in response to an eccentricity stimulus for the effective connectivity study. (**A**) (V1), (**B**) (V2), (**C**) (V3), (**D**) (Vp), (**E**) (V3a), (**F**) (V4v), (**G**) (V3b), and (**H**) (V5/MT) were included in the brain network. The dotted curve shows the fMRI response, while the red curve indicates the input from the experimental design. The *x*-axis is the normalised fMRI signal, while the *y*-axis is time (in terms of TR = 3 s).

The brain response to the eccentricity stimulus was adopted to validate the method in this example. Based on the boundaries from the region of the visual cortex, one fMRI time series from each visual cortex region was selected randomly. For the eight-connection brain visual system (Figure 1B), the fMRI response or system output time series is displayed as the dotted asterisk curves in Figure 2. The red curve is the input for the time series, i.e., it assumes that the system input contributes to generate an fMRI response. The red curve is the fundamental frequency of the fMRI response, which was obtained from the fast Fourier transform (FFT) to model the system input.

**Figure 3:**
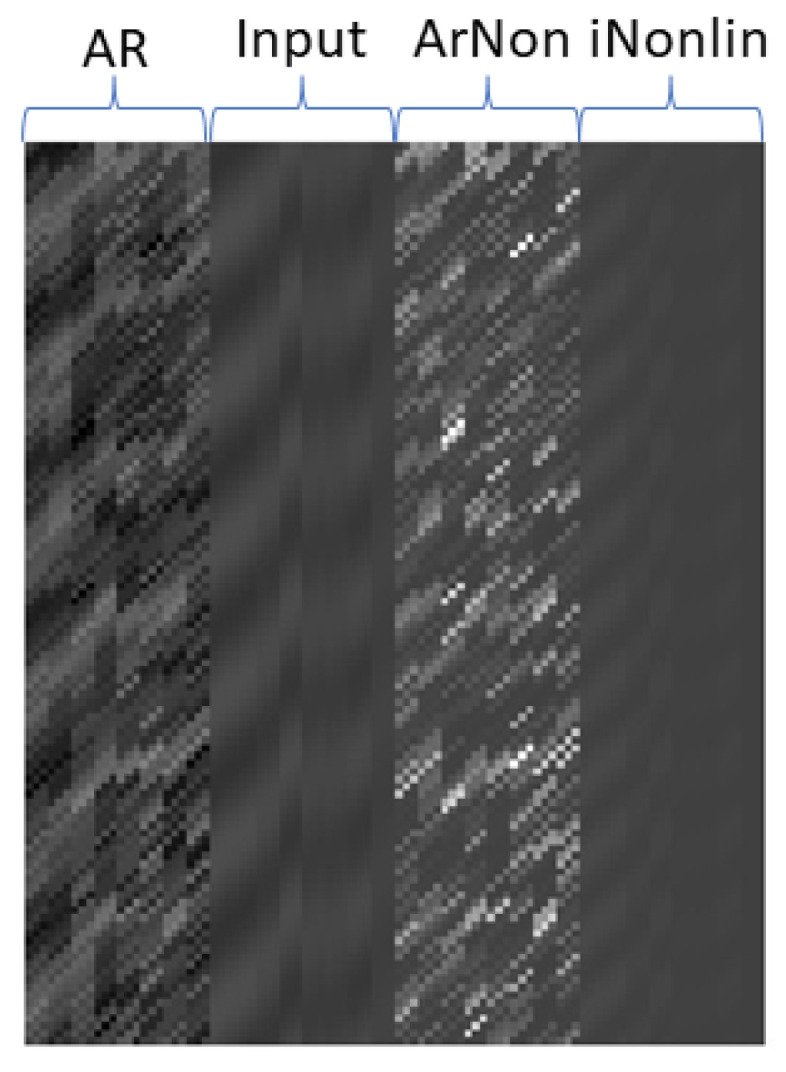
Predictors matrix, e.g., $x_{i}^{T}$ in Equation (10).

Based on the response as shown in Figure 2, we can generate the polynomials matrix (Equations (9) and (10)) as shown in Figure 3 for the connectivity study. As the AR is three-order (AR = 3), the matrix has the dimension of (120-3) × 96 (excluding the intercept).

From Figure 2, we can generate a predictor matrix as shown in Figure 3. In the Figure, the first 24 columns of the matrix (denoted as AR on the top) are derived from an auto-regression term from different regions, e.g., $Y_{1}\left(t-1\right),Y_{2}\left(t-1\right),\cdots ,Y_{8}\left(t-3\right)$. The second 24 columns (“Input” on the top) are the inputs for different regions, e.g., $u_{1}\left(t-1\right),u_{2}\left(t-1\right),\cdots ,u_{8}\left(t-3\right)$. The third 24 columns (“ArNon” on the top) are nonlinear terms of the responses, e.g., $u_{1}^{2}\left(t-1\right),u_{2}^{2}\left(t-1\right),\cdots ,u_{8}^{2}\left(t-3\right)$. The last 24 columns (denoted by “iNonlin”) are the nonlinear terms of inputs from eight-connected regions, e.g., $Y_{1}^{2}\left(t-1\right),Y_{2}^{2}\left(t-1\right),\cdots ,Y_{8}^{2}\left(t-3\right)$.

**Figure 4:**
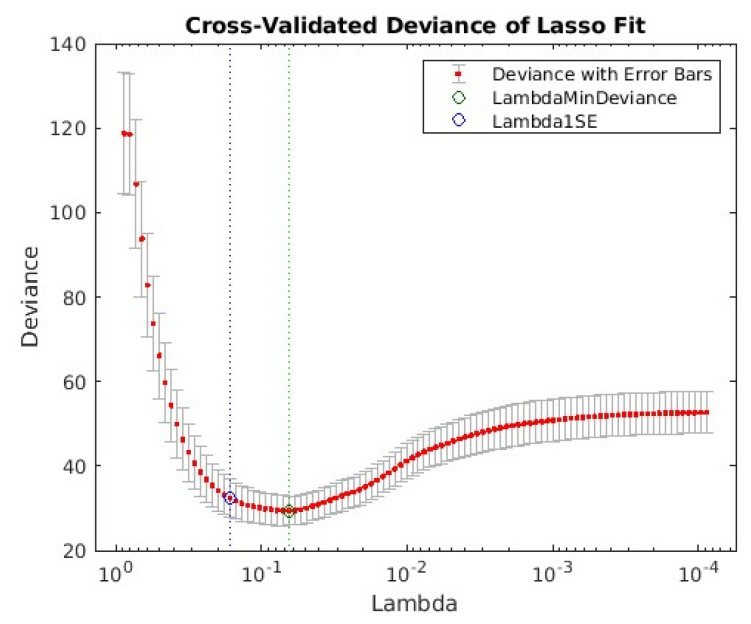
LASSO selection results. The blue circle and (**left**) dotted line locate the point with the minimum cross-validation error plus one standard deviation (Lambda 1SE = 0.1643). The green circle and (**right**) dotted line locate the Lambda with minimum cross-validation error (LambdaMinDeviance = 0.0648).

Resolving Equation (10) by minimizing Equation (11), we obtained the L1 regularization parameter Lambda for model selection. The selection process with the 10-fold cross-validation method is illustrated in Figure 4.

In Figure 4, there are two vertical dashed lines. The vertical dashed line on the right (green) corresponds to the Lambda for the minimum mean cross-validated error. The vertical dotted dashed line on the left (blue) locates the point with a minimum cross-validation error plus one standard deviation (Lambda = 0.1643). Lambda with one standard error (1SE) is the value that gives the most regularised model such that the cross-validated error is within one standard error of the minimum. In this study, the Lambda value corresponding to the minimum deviance was adopted for LASSO model selection.

For the model as described in Equation (9), and the responses shown in Figure 2, applying the LASSO method, the selected model $y_{1}\left(t\right)$ for the response is:

(12) $$y_{1}\left(t\right)=-0.0735-0.0121y_{3}\left(t-3\right)+6.4472u_{3}\left(t-2\right)+3.9456u_{3}\left(t-3\right)+0.019y_{4}^{2}\left(t-1\right)+0.3069u_{3}^{2}\left(t-2\right)+\varepsilon$$

Five items have been selected in Equation (12), and the associated coefficients are displayed. Applying Equations (7) and (8), we obtained the *t*-test value (the degree of freedom is N−k = 117 − 6 = 111) as 1.6383 (intercept), 0.9175 ($y_{3}\left(t-3\right)$), 12.7305 ($u_{3}\left(t-2\right)$), 8.2911 ($u_{3}\left(t-3\right)$), 1.4245 $y_{4}^{2}\left(t-1\right)$, and 2.1506 $u_{3}^{2}\left(t-2\right)$. From the t table, the influence from $u_{3}\left(t-2\right)$, $u_{3}\left(t-3\right)$, and $u_{3}^{2}\left(t-2\right)$ to $y_{1}\left(t\right)$ was significant (*p* < 0.05).

**Figure 5:**
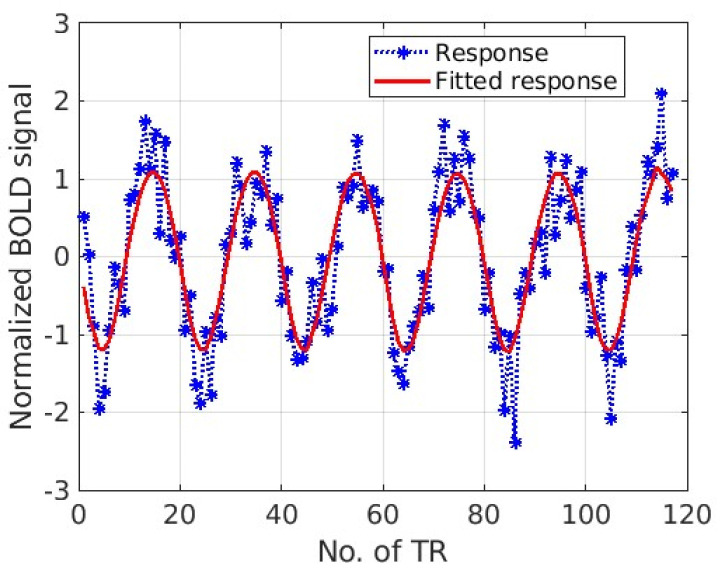
A visual cortex (V1 region) response to the stimuli and final prediction results from the identified model structure and its parameters. The red curve shows the prediction result from the V1 region.

Based on Equation (12), we can plot the final prediction result as shown in Figure 5. The dotted blue curve is the response from V1, while the red curve is the fitted results with the LASSO model response from Equation (12). Another validation study example of an effective connectivity study in the visual cortex with polar angle stimuli is presented in the Appendix A.

## 4. Discussion

Nonlinear system identification methods [5] and MAR methods [7] have been extensively suggested for modelling dynamic brain systems. Technically, the main difference between these two methods is that for the MAR method, system inputs and nonlinear terms are not considered for modelling the dynamic system. For the nonlinear system identification method, both nonlinear terms and system inputs can be included in the model. For the nonlinear system identification method, other kernels such as radial basis functions can also be applied to model the system [31]. While the MAR method is a special case for nonlinear system identification methods, if the identification kernel is polynomial and without system input, and there are nonlinear terms in the model for selection, the MAR method is the same as the identification method [2].

Stimuli from experimental design can be regarded as a system input as it is something put into the brain system or expended in its operation to achieve output. Statistical model selection was employed to decide whether the response was caused by system inputs (stimuli) or the connected regions. In a block design with periodic stimuli, including the phase-encoding block design as shown in this study, the input is a travelling square wave, which has delays, and the shape has been distorted. Therefore, the fundamental frequency of the response was adopted for a representative system input [3]. For resting-state fMRI studies, it can be regarded as a special case, i.e., a system without input or the input is 0.

In addition to the LASSO method, the last-angle regression (LARS) method for model selection has been applied for selecting models to study effective connectivity [20]. The LARS algorithm is a greedy method that does not yield a provably consistent estimator. The LASSO method has been applied to functional and effective connectivity studies [10,27], while these methods are based on MAR models. Stepwise regression, backwards selection such as recursive feature elimination (RFE), and elastic net regularisation methods [16] can be applied to identify the structure of the brain connection networks.

Individual regional effective connectivity variability is bigger when applying the model selection method for causality inference (comparing the selected model in the Section 3 and Appendix A). This is because the model selection is sensitive to the fMRI response. The fMRI image spatial resolution in this study is 4 mm × 4 mm × 4 mm. A single voxel in fMRI measures the response of a neural population, which includes the response from millions of single neurons. The response at each voxel is different, so the causality inference based on each voxel is different accordingly. A small change in an fMRI response will lead to a bigger change in effective connectivity. Furthermore, when applying the LASSO method for model selection, a ten-fold cross-validation method was used, which also has randomness.

The limitation of this study is that the method has not been validated on an event-related fMRI response. In this study, phase-encoded experiments were adopted. This experimental design can be regarded as a travelling wave, and it is similar to a block design. The advantage of this type of experimental design is that the Fourier transform can be applied to approximate the system input. This makes the analysis simpler but more powerful. For the other types of experimental design, system inputs are much more difficult to model, and the system input needs to be adjusted to fit the study.

Compared with other studies [10,27], the advantage of this study is that this method takes nonlinear and system inputs into account for modelling the effective connectivities. In addition, this study applied LASSO methods for identifying the system structure and associated parameters automatically. This study combined the results from model selection with causality inference and was tested in a real human visual system.

One of the future directions for applying the results of this study is to overcome the limitations of the randomness for the causality study. Statistical methods need to be developed to address this problem. At the subject level, there are individual variabilities for measuring causality. Methods need to be developed to deal with individual variabilities and combine different causality results with statistical measurements for group analysis. To reduce the model randomness, robust statistical methods need to be applied to detect the outliers from the estimation.

Furthermore, a deep learning method has been proposed to model the nonlinear system [32]. In the framework for system identification, an autoencoder structure of the neuron network was introduced, in which an encoder was employed to estimate the deep features (latent variables) of the model. Then a decoder was applied to restore the system output estimation. Using deep features, the causality relationship between different deep features could be inferred.

Finally, this study used the fMRI from the visual cortex. Other cortexes and other stimuli have not been verified. For example, it is possible to apply this method to study the motor cortex system causality relationship. Additionally, it may be of interest to apply this method to study the human brain under disease conditions. Lastly, similar to a dynamic functional connectivity study that adopted sliding windows [33], this method can be extended to use the sliding windows method to study dynamic casualty.

## Figures and Tables

**Figure A1:**
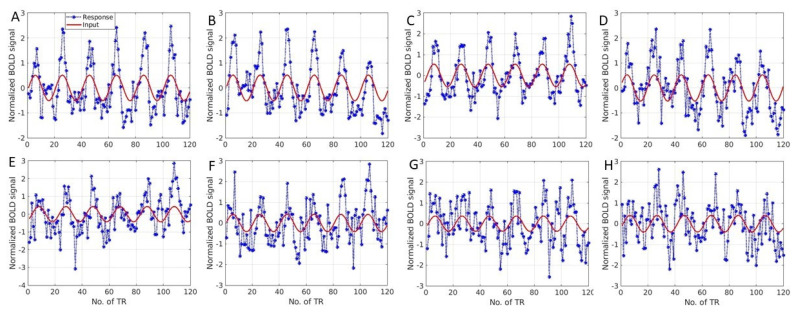
Time series from the eight-connection visual system in response to polar angle stimulus for effective connectivity study. The fMRI response was obtained from (**A**) (V1), (**B**) (V2), (**C**) (V3), (**D**) (Vp), (**E**) (V3a), (**F**) (V4v), (**G**) (V3b), and (**H**) (V5/MT). The dotted curve shows the fMRI response, while the red curve indicates the input.

**Figure A2:**
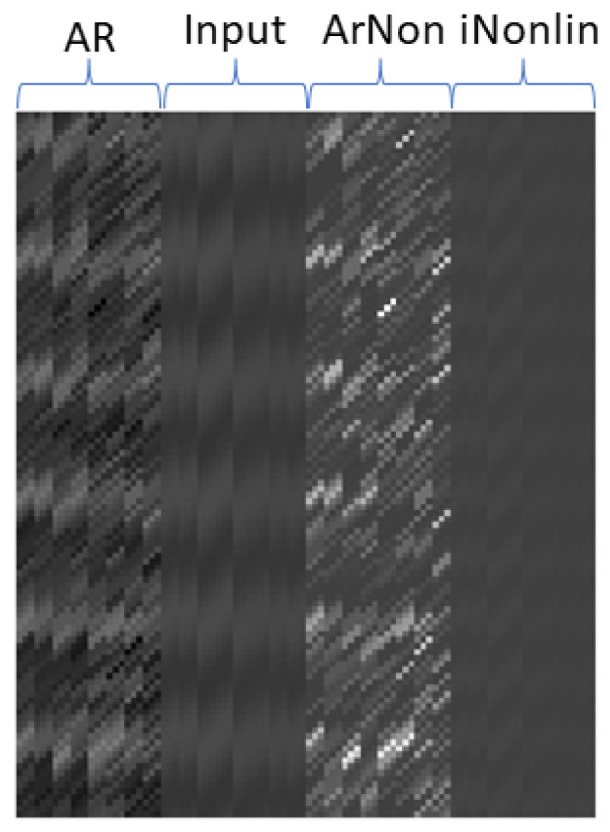
Predictor matrix, i.e., $x_{i}^{T}$ in Equation (10).

**Figure A3:**
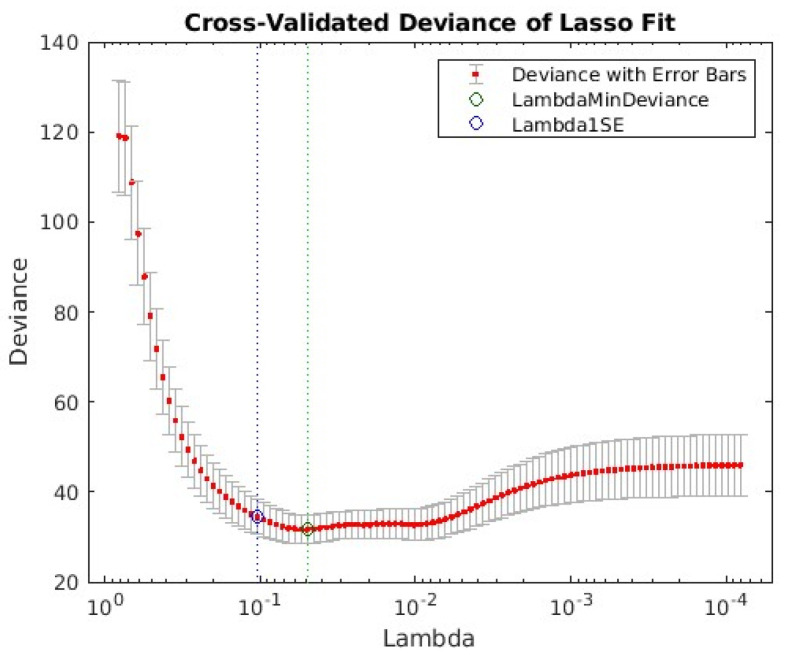
LASSO selection results and Lambda values. Lambda 1SE = 0.1045 is the regularisation corresponding to the minimum cross-validation error plus one standard deviation. The vertical dotted line on the right indicates the minimum cross-validation error value (Lambda MinDeviance = 0.0497).

**Figure A4:**
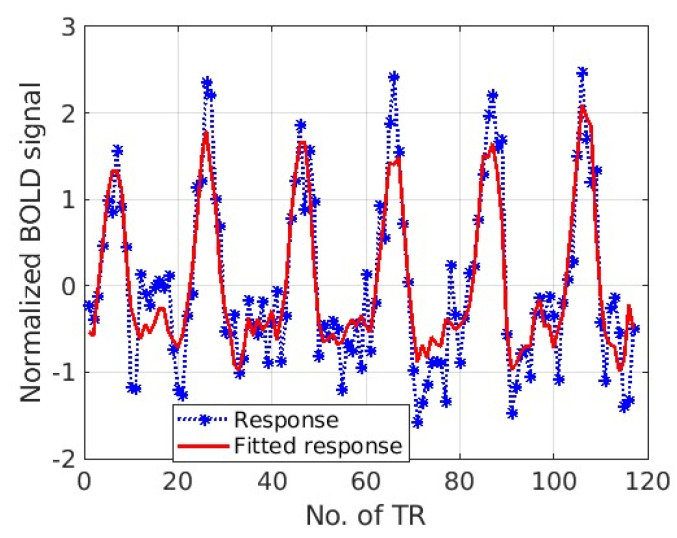
The final prediction result based on the identified structure and its parameters. The dotted curve shows the fMRI response, while the red curve indicates the predicted response for the LASSO selected model. The *x*-axis is the magnitude of the normalized fMRI response, while the *y*-axis is time in terms of TR.

## Data Availability

The original contributions presented in the study are included in the article (https://doi.org/10.1167/iovs.06-1021) Further inquiries can be directed to the corresponding authors.

## Appendix A

In this appendix, effective connectivity at the same voxel in response to polar angle stimuli viewing with the right eye from the same subject was employed in the analysis. The fMRI response in V1 (A), V2 (B), V3 (C), Vp (D), V3a (E)), V4v (F), V3b (G), and V5/MT (H) is displayed in Figure A1. Compared with Figure 2, the fMRI response is very different, particularly in the V5/MT region.

Similar to Figure 3, Figure A2 shows the predictor matrix obtained from fMRI responses (Figure A1). In the figure, the first 24 columns of the matrix (denoted as AR on the top) are derived from auto-regression terms from different visual cortex regions. The second 24 columns (“Input” on the top) are the inputs for different regions. The third 24 columns (“ArNon” on the top) are nonlinear terms of the responses, and the last 24 columns (denoted by “iNonlin”) are the nonlinear terms of inputs from the eight-connected visual cortical regions.

Resolving Equation (10) by minimising Equation (11), we obtained Lambda from the LASSO method. The selection process is illustrated as shown in Figure A3.

In Figure A3, there are two vertical dashed lines. The vertical dashed line on the left (blue) corresponds to the lambda with minimum cross-validation error plus one standard deviation (Lambda 1SE = 0.1045). The vertical dashed line on the right (green) locates the Lambda with minimum cross-validation error (Lambda = 0.0497). The Lambda with minimum deviance value was adopted for model selection in this study.

For the model as described in Equation (9), and the responses as shown in Figure A1, applying the LASSO method, the selected model $y_{1}\left(t\right)$ for response is:

$$y_{1}\left(t\right)=0.0376+0.1819y_{1}\left(t-1\right)+0.0904y_{2}\left(t-1\right)+0.0618y_{3}\left(t-1\right)+\;0.2021y_{3}\left(t-2\right)+0.0219y_{5}\left(t-3\right)+0.0581y_{8}\left(t-2\right)+0.1512u_{3}\left(t-1\right)+\;0.4077u_{5}\left(t-2\right)+{0.0560y}_{3}^{2}\left(t-1\right)+{0.0343y}_{3}^{2}\left(t-2\right)+0.0237y_{3}^{2}\left(t-3\right)-\;0.0069y_{4}^{2}\left(t-2\right)+0.0816y_{7}^{2}\left(t-2\right)+0.0962u_{3}^{2}\left(t-1\right)-1.6881u_{4}^{2}\left(t-3\right)+\varepsilon .$$

Sixteen items have been selected in the above equation, and the associated coefficients were also included. Applying Equations (7) and (8), we obtained the T test value (degree of freedom is N−k = 117 − 6 = 111) for each coefficient as 0.7835 (intercept), 4.7356, 2.7119, 2.0795, 5.1189, 1.0681,1.9739, 2.0008, 3.4942, 2.5979, 1.8091, 1.4014, 0.6195, 3.2746, 0.7606, and 6.6170, respectively. From the t statistic table, the influence from $y_{2}\left(t-1\right)$ and $y_{3}\left(t-1\right)$ to y1 is significant (*p* < 0.05). Based on the selected model, the prediction of the response (red curve) is displayed in Figure A4.
